# Summarizing the Advantages of the Intraflap versus Cranial–Caudal Anastomoses in Stacked Free Flap Breast Reconstruction

**DOI:** 10.1055/a-2321-6279

**Published:** 2024-06-10

**Authors:** Alberto Franchi, Luca Patanè, Bettina Gögh, Florian Jung, Abdul Rahman Jandali

**Affiliations:** 1 Department of Hand- and Plastic Surgery, Cantonal Hospital of Winterthur, Winterthur, Switzerland; 2 Department of Surgery, Plastic Surgery Unit, Sapienza University of Rome, Rome, Italy

Getting enough volume in autologous breast reconstruction can be difficult and transferring multiple free flaps to reconstruct a single breast can provide a solution to that problem. How to connect the free flaps pedicles to the recipient site has been a point of discussion in the literature and two main approaches have been described. The first one involves using the caudal stumps of the internal mammary (IM) vessels and is usually referred to “cranial–caudal” approach. The second one implies the anastomosis between one flap's pedicle to a branch of the other one. 1 This technique has been named in different ways in the literature: intraflap, flow-through, daisy-chain, chain-link, and others.

In the present letter, we want to list what we think are all the advantages of the intraflap approach compared with the cranial–caudal one. To our knowledge, some of them have not been mentioned in the available literature.

**Fig. 1:**
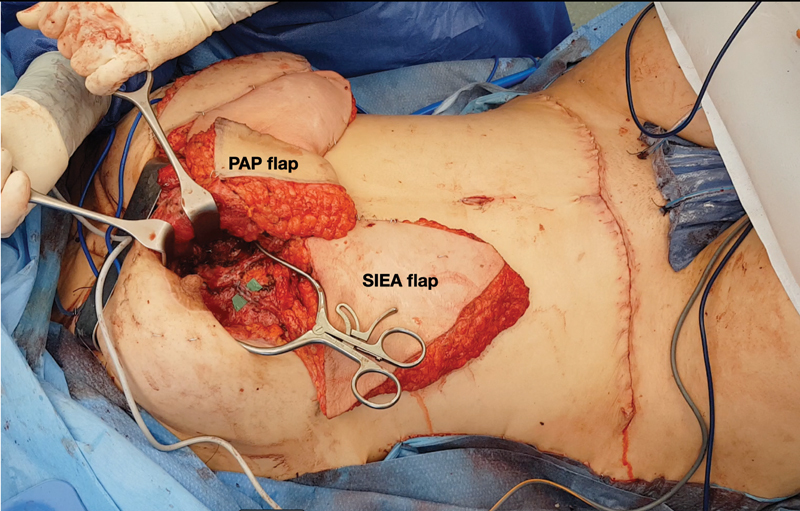
A case of bilateral autologous breast reconstruction using stacked flaps. The PAP flap is anastomosed to the IM vessel using a rib-sparing approach and the SIEA flap is anastomosed to a branch of the PAP flap. The first flap (PAP) can be completely deepithelialized and buried to give volume and projection. The second flap (SIEA) will reconstruct the missing lower pole and serve to monitor the perfusion postoperatively. A functioning second flap implies functioning anastomoses of the first flap. IM, internal mammary; PAP, profunda artery perforator; SIEA, superficial inferior epigastric artery.

**Fig. 2:**
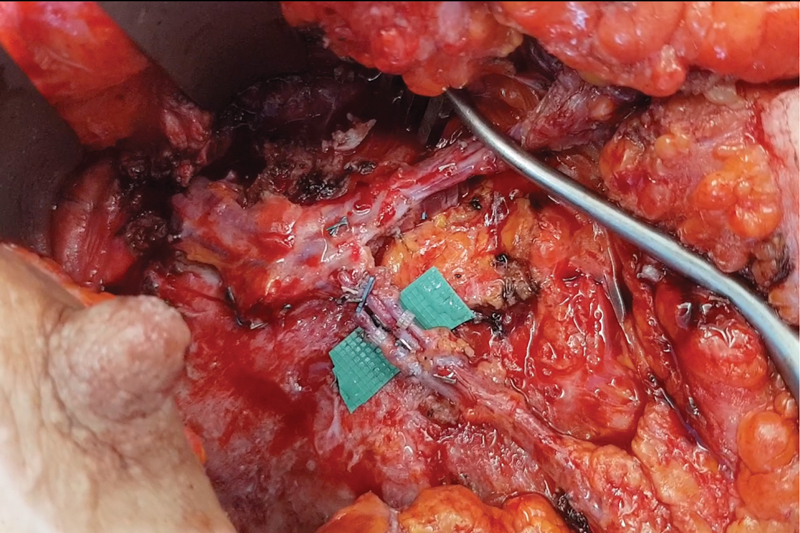
Closeup picture of the intraflap anastomoses of the SIEA pedicle to the PAP pedicle. Two veins and one artery are shown on the green background. Note how the intraflap anastomoses can be performed on the chest wall in a more comfortable, more external position if compared with the anastomoses to the IM vessels that are situated deeper in a narrower space. IM, internal mammary; PAP, profunda artery perforator; SIEA, superficial inferior epigastric artery.

**Fig. 3:**
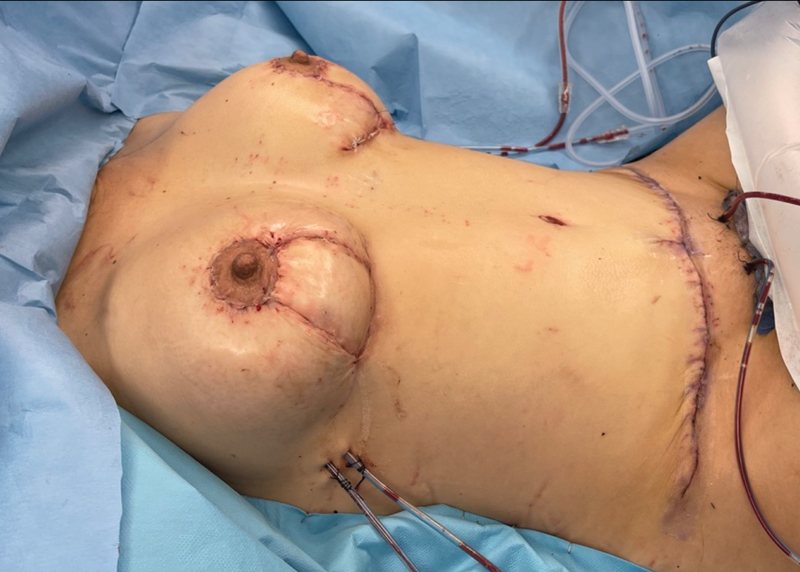
Final intraoperative picture. The external flaps are used to reconstruct the lower pole and serve for postoperative monitoring.

1. In the intraflap technique, the anastomoses of the first flap can be performed to a perforator of the IM vessels (when sizeable) or to the IM vessels through a rib-sparing approach ( Figs. 1 and 2 ). Since the second flap is anastomosed to the first, there is no need for a wide exposure of the IM vessels as required in the cranial–caudal technique. Reducing the damage to the intercostal musculature and ribs lowers the postoperative pain and risk of parasternal hollowing. The pedicles of the flaps mostly used for autologous breast reconstruction (e.g., deep inferior epigastic artery perforator and profunda artery perforator) usually offer some sizeable branches that can be used for the intraflap technique and the branch with the most appropriate length and best caliber match to the second flap's pedicle can be chosen. However, the caliber discrepancy or small caliber of the available branches for the anastomoses can pose a technical challenge to the less experienced surgeons.
2. The intraflap anastomosis of the second flap to the first can be performed in a more comfortable, more “external” position for the surgeon ( Figs. 1 and 2 ) compared with the cranial–caudal technique where the microsuturing is performed in a deeper and more limited space. Alternatively, the intraflap anastomoses between the two flaps can be performed on a side table before transferring the construct (technically a prefabricated chimeric flap) to the chest and performing the anastomosis to the IM vessels.
3. If the first flap (the one anastomosed to the IM vessels) is completely deepithelialized and buried, just the second “external” flap needs to be monitored. In fact, a good perfusion of the second flap implies patent anastomoses of the buried one. This is shown in Fig. 3 (the patient signed informed consent to publish her photo). In the cranial–caudal anastomosis, if one flap is completely deepithelialized and buried, the postoperative monitoring is not possible or requires special monitoring devices (e.g., flow coupler).
4. The quality of IM vessels in previously irradiated patient can be poor and the anastomosis can be challenging. Using the intraflap technique reduces the number of anastomoses to the damaged IM vessels. Moreover, if the IM vessels are unusable for any reason, the intraflap technique allows a double flap reconstruction using another recipient pedicle (such as the subscapular system where a cranio–caudal approach is not possible/has never been described).
5. The IM vein will often bifurcate at the third to fourth intercostal space, thus rendering the caudal venous anastomosis more challenging. The overall reliability of the retrograde IM vessels has been questioned by several authors. 2 3

It must be mentioned that in the intraflap technique, an anastomotic failure of the first flap at the IM vessels would put at risk both flaps, thus constituting a disadvantage over the cranial–caudal technique.

**Fig. 4:**
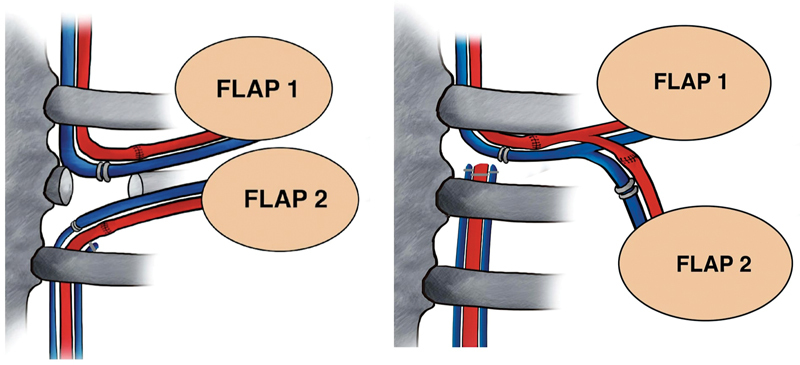
Schematic representation of the two techniques.

Another advantage of the cranial–caudal approach is the surgeon's convenience of having a single vascular axis (the IM artery and vein) as recipient for both flaps pedicles' anastomoses. The preparation of the vessels and the microsurgical suturing can be performed under the microscope in a single microsurgical field without changing the microscope and surgeon's position. Fig. 4 helps visualize some of the listed advantages and disadvantages of the two techniques.

Having taken all these considerations into account we favor the intraflap technique and do not perform the cranio–caudal approach anymore.
